# Compliance with infection prevention and control standard precautions and associated factors among healthcare workers in four health facilities in Fako division, Cameroon

**DOI:** 10.1186/s12913-025-12594-z

**Published:** 2025-04-24

**Authors:** Midrelle Syntyche Tsague, Leslie Tasha Mbapah, Denis Georges Teuwafeu, Fombo Enjeh Jabbossung, Sandra Tabe Etaka, Ngwa Fred Ngunjoh, Aghinwi Brandon Forcob, Longsti Scarlet Tabot Enanga, Brandon Carl Monika Pouekoua, Nicholas Tendongfor

**Affiliations:** 1 Faculty of Health Sciences, University of Buea, Buea, Cameroon; 2 Triad Research Foundation (TRF), Buea, Cameroon

**Keywords:** Healthcare-associated infection, Infection prevention and control, Standard precaution, Healthcare workers, Compliance, Health facilities, Cameroon

## Abstract

**Background:**

Healthcare-associated infections (HAI) are a serious public health problem. Healthcare workers are exposed to HAI, which in turn exposes patients to nosocomial infection. Compliance with infection prevention and control (IPC) measures can help break the infection chain and halt the transmission of infection to healthcare workers and patients. There is a paucity of evidence-based data on the level of compliance of healthcare workers (HCWs) with IPC in the Fako division. This study assessed healthcare workers’ compliance with infection prevention and control standard precaution measures and its associated factors, in Fako division, Cameroon.

**Methods:**

A hospital-based cross-sectional study was conducted in four health facilities in the Fako division of Cameroon. A standardised observation checklist and a validated questionnaire were used to assess healthcare provider compliance with standard precautions for the prevention of infection. Data was analysed using StataMP 18.0. A multivariable logistic regression analysis was used to identify independent factors associated with compliance with infection prevention control (IPC) measures.

**Results:**

We recruited 276 participants, and the Overall compliance with the IPC was 64.5%. Laboratory technicians had a compliance to IPC proportion of 91.7%, nurses 62.4%, doctors 60.9%, and midwives 45.8%. Professional Cadre [aOR = 8.32 (95% CI: 1.90–36.53), *P* = 0.005], health facility [aOR = 3.61 (95% CI; 1.29–10.07), *P* = 0.014], and the need for transmission-based precaution [aOR = 2.41 (95%: 1.38–4.19), *P* = 0.002] were independently associated with good compliance with IPC measures.

**Conclusion:**

Compliance with infection prevention control standard precaution measures of HCWs in the Fako Division was suboptimal and varied according to professional qualifications, health facilities, and departments. Factors associated with good compliance with IPC measures have been identified. These findings highlight the need for hospital-based interventions to improve HCW compliance with IP measures and break the infection transmission chain in the hospitals.

**Supplementary Information:**

The online version contains supplementary material available at 10.1186/s12913-025-12594-z.

## Background

Healthcare-associated infection (HAI) increases morbidity and mortality, extends the length of hospital stay, fuels the emergence of antimicrobial resistance, and raises healthcare costs for patients and providers [1]. In high-income countries, HAI affects about 7% of patients, meanwhile, in low and middle-income countries (LMICs), it is as high as 15% [2]. A study conducted in 2016 at the Yaoundé Teaching Hospital in Cameroon showed that the incidence of HAI was 19.25%, with a mortality rate of 28% [3]. Healthcare workers (HCWs), due to the nature of their work, are exposed to body fluids, and the World Health Organization(WHO) estimates that about 2.5% of HIV cases and 40% of Hepatitis B and C cases among HCWs are the result of these exposures [4]. The WHO estimated that about 3 million HCWs are exposed to blood-borne viruses each year, and 90% of the exposures occur in LMICs [5]. Good infection prevention control programs can aid in decreasing the burden of HAI by 70% [6].

Infection Prevention and Control (IPC) refers to event-based practices and methods likely to prevent or reduce the transmission risk of microorganisms to healthcare providers, other patients, hospitalised patients, and visitors if they are evenly applied in healthcare structures [7]. The two main components of IPC are standard precautions and complementary precautions.

Standard Precautions or Universal Precautions (UP) are a standard set of guidelines to prevent the transmission of bloodborne pathogens and other potentially infectious materials [8]. This includes practicing hand hygiene and using personal protective equipment (PPE) such as gowns, gloves, masks, and face shields or goggles, as they serve as a barrier to protect the skin, mucous membranes, airway, and clothing.

To prevent the spread of infections, HCWs need to comply with IPC. However, several observational studies have shown limited adherence to recommended practices by healthcare personnel [9]. This problem with noncompliance is significant because more than 6 million HCWs are at risk, and there is a 0.3% risk of infection with HIV after percutaneous exposure to HIV-contaminated blood [10]. Despite these glaring problems, there is a paucity of evidence-based data on the level of compliance of HCWs with standard precautions (SPs) in these settings. The absence of data makes it difficult to advocate for a positive change.

## Materials and methods

### Aim

This study sought to close the data gap in the Fako Division by assessing the compliance of HCWs with infection prevention and control measures (standard precautions) and identifying the factors associated with HCWs’ compliance level with these measures.

### Study design and setting

A hospital-based cross-sectional study was conducted over 5 months (1st January to 31st May 2024) among HCWs from four health facilities (two public and two private) in Fako Division. The four hospitals include Hospital #1 and Hospital #2 which are government-funded and are the region’s two main referral and teaching hospitals, whereas Hospital #3 and Hospital #4 are private district-level facilities. They were conveniently selected based on the nature of funding, high capacity, and patient turnout.

Hospital #1 is a secondary health facility and a main referral hospital in the Southwest Region. The hospital is made up of four major departments, which include: Pediatrics, Internal Medicine, Surgery, Obstetrics, and Gynecology (OBGYN). The hospital also has specialized centres such as the Dialysis Centre, Intensive Care Unit (ICU), Ophthalmology Unit, Dentistry, Medical Imaging, Neonatology, and kangaroo Mother Care (KMC). The healthcare workers are made up of doctors (30, nurses and midwives (180), laboratory technicians (lab. technician)(30), and pharmacy attendants(06). The hospital has a sanitation department but no statutory meetings.

Hospital #2 serves as a secondary health facility and a main referral hospital located in the central town of Limbe. The hospital has a Pediatric department, OBGYN department, Internal Medicine department, Surgical department, Dentistry, Ophthalmology, Physiotherapy, and Intensive Care Units. It has an Imaging Centre, two Theatres, and an equipped Laboratory. The healthcare workers are made up of doctors (39), nurses and midwives (181), lab. technicians (36), and pharmacy attendants (06). The hospital has an IPC committee with neither a specified meeting period nor regular follow-up.

Hospital #3 is situated in Buea at the foot of Mount Cameroon. The total catchment area is about 50,000. It has Internal Medicine, Pediatric, Maternity, Laboratory, and Outpatient units. The HCWs are made up of doctors (05), nurses (26), midwives (13), lab. technicians (09), and pharmacy attendants (02). Hospital 3 has an IPC committee with regular monthly meetings with staff.

Hospital #4 is located in the Buea, it is a private clinic. The clinic is made up of Medical, Surgical, Maternity, Pediatric, and Laboratory units. The HCWs are made up of doctors (9), nurses (27), midwives (09), lab. technician (08), pharmacy (05). In the clinic, the IPC committee is an ad hoc committee where they convene when needed.

### Study population

All healthcare workers working within the four aforementioned health facilities for at least six months and who gave informed consent were included in the study. These HCWs included medical doctors, nurses, midwives, and laboratory scientists. A total number of 276 participants were included in the study using Yamane’s formula as shown below

$$\:\varvec{n}=\frac{\varvec{N}}{\varvec{1}+\varvec{Ne}^{\varvec{\wedge}}2}$$

n = Minimum sample size

N = Total number of functional HCWs in all four hospitals = 607

e = precision at 0.05 at a 95% confidence interval.

Minimum calculated sample = 242 HCWs. We considered 267 as our minimum sample size to account for a 10% non-response rate.

The participants were recruited using sampling proportionate to size from each of the four facilities as shown: $\:\frac{\varvec{n}\times\:\varvec{N}\varvec{f}}{\varvec{N}}$

n: minimum sample size of the study population

Nf: total number of healthcare workers in the health facility

N: the total number of healthcare workers in all the hospitals

Hospital #1 minimum sample population: $\:\frac{267\times\:240}{607}=105.56\sim106$

Hospital #2 minimum sample population: $\:\frac{267\times\:256}{607}=\;112.60\;\sim\;113$

Hospital #3 minimum sample population: $\:\frac{267\times\:53}{607}=23.31\sim24$

Hospital #4 minimum sample population: $\:\frac{267\times\:58}{607}\;=25.51\sim26$

### Data collection

The data collection tool comprised a structured questionnaire and an observation sheet. The structured questionnaire was adapted from existing literature and similar studies. It contained information on sociodemographic characteristics, knowledge of IPC (made up of a set of 10 structured questions that delved into IPC-related topics), and IPC-related characteristics [11–13]. The observation sheet was adapted from the WHO hand hygiene observation form [14] (see Additional file 1). The data collection form was pre-tested in a different facility and modified accordingly.

The HCWs were met on duty, with informed written consent obtained 24 h before administering the data collection form to reduce the Hawthorn effect. Each participant was observed only once for at least 20 min when caring for a patient or carrying out a diagnostic procedure. Three opportunities for IPC compliance measures to be implemented were recorded per participant. The observer noted opportunities for the need for precautionary measures, the indication, and whether action was taken or not, with emphasis on the WHO five-period for hand hygiene. This was done only in the observer’s field of view (patient care area to be observed and includes visible areas where HCW can clean the hands e.g. sinks and standby alcohol dispensers which varied based on the structure of the facility) defined before the start of the observation. If the HCW left the field of view without taking any action, it was considered that the HCW never did. This observation was done per HCW per department, covering workers on day and night shifts.

After this observation, a structured questionnaire with two parts: Part 1, with a score from 0 to 10, was immediately self-administered to collect socio-demographic and IPC-related characteristics and to assess their knowledge of infection prevention and control practices (10-item question) [15] (see Additional file 2).

### Data analysis

Data was verified, entered into the data collection form designed on Kobo Collect, and exported to Excel 2016 for cleaning. All participants’ information was coded to ensure confidentiality.

Data cleaned in Excel was exported into StataMP 18.0 for analysis. The data was explored to identify hidden patterns and important variables. Categorical variables were presented as frequencies and percentages, quantitative variables as means with standard deviation (SD), or median with interquartile range after checking for normality of distribution. A cut-off for good compliance was set at an overall score of ≥ 80% according to the compliance standard precautions scale (CSPS) (Lam SC: Compliance with standard precautions scale: fact sheet, unpublished). A good knowledge level was defined as a knowledge score ≥ 7/10 since the mean score was 7.0. The overall compliance proportion was calculated as the total HCWs with ≥ 80% compliance level. This was also calculated and reported by cadre, facility, and department. The Chi-square test was used to compare proportions. Multivariable logistic regression analysis with backward elimination was used to identify factors independently associated with good compliance. Multicollinearity was checked with the mean-variance inflation factor (VIF) = 1.19 and the model fitness with Pearson’s goodness of fit (*p* = 0.30). The likelihood ratio *p*-values were reported with their adjusted odd ratios and 95% confidence intervals. The level of significance was set at *p*-value < 0.05.

## Results

### General characteristics of the participants

In this study, we recruited 276 participants, and the mean age was 26.3 (± SD 6.0) years, with 18–25 years being the most represented age group. Most participants were female, 72.8% (*n* = 201), and the majority of participants were from public facilities (Table 1).

**Table 1:** Sociodemographic characteristics of healthcare workers in four health facilities in the Fako division, Cameroon

**Variable**	**Frequency, n**	**Percentage, %**
**Gender**		
Male	75	27.2
Female	201	72.8
**Age ± SD (years)**	26.3 ± 6.0	
18 to ≤ 25	226	81.9
> 25 to ≤ 35	41	14.9
> 35	9	3.2
**Cadre**		
Doctor	69	25.0
Lab. Technician	36	13.0
Midwife	24	8.7
Nurse	147	53.3
**Facility**		
Buea Regional Hospital	74	26.8
Limbe Regional Hospital	117	42.4
Mount Mary Hospital	58	21.0
Solidarity Hospital	27	9.8
**Type of facility**		
Public	198	71.7
Private	78	28.3
**Department**		
Laboratory	37	13.4
Medical	59	21.4
OBGYN	41	14.9
Outpatient	37	13.4
Pediatric	29	10.5
Private ward	11	4.0
Surgical	62	22.5
**Work status**		
Contract	66	23.9
State worker	29	10.5
Volunteer	181	65.6
**Work Shift**		
Day	174	63.0
Night	102	37.0
**Years of Practice**		
< 3 years	84	30.4
3 - 7 years	167	60.5

*OBGYN* Obstetrics and gynaecology, *Lab*. *technician *Laboratory technician, *% *Percentage, *SD *Standard Deviation

### IPC-related characteristics among study participants

In this study, 34.8% (*n* = 96) had good knowledge of IPC, 87.3% (*n* = 241) of participants had personal protective equipment available in their health facilities, 87% (*n* = 240) had IPC guidelines present in their department, 67.7% (*n* = 187) had received IPC training, and 69.9% (*n* = 193) had IPC committees present in the facility. Concerning the infrastructure, 85.1% (*n* = 235) of participants had a constant water supply in the facility, and 80.8% (*n* = 223) had a constant electricity supply in the facility (Table 2).

**Table 2:** Infection prevention and control-related characteristics among health care workers in four health facilities in Fako division, Cameroon

**Variable**	**Frequency, n**	**Percentage,%**
**Personal protective equipment available**		
Yes	241	87.3
No	35	12.7
**IPC guidelines present in the department**		
Yes	240	87.0
No	36	13.0
**IPC training**		
Yes	187	67.7
No	89	32.3
**IPC committee present in the facility**		
Yes	193	69.9
No	83	30.1
**Hepatitis B vaccination**		
Yes	186	67.4
No	90	32.6
**Covid-19 vaccination**		
Yes	186	32.6
No	90	67.4
**Constant water supply in the facility**		
Yes	235	85.1
No	41	14.9
**Constant electricity supply in the facility**		
Yes	223	80.8
No	53	19.2
**Adequate handwashing points in the department**		
Yes	245	88.8
No	31	11.2
**Transmission-based precaution indicated**		
Yes	142	51.4
No	134	48.6

*IPC *Infection prevention and control, *TBP *Transmission-based precaution, *% *Percentage

### Compliance with IPC

Overall compliance with the IPC was **64.5% (95% CI: 58.5–70.1)**.

### Compliance with IPC by health facility

**Fig. 1:**
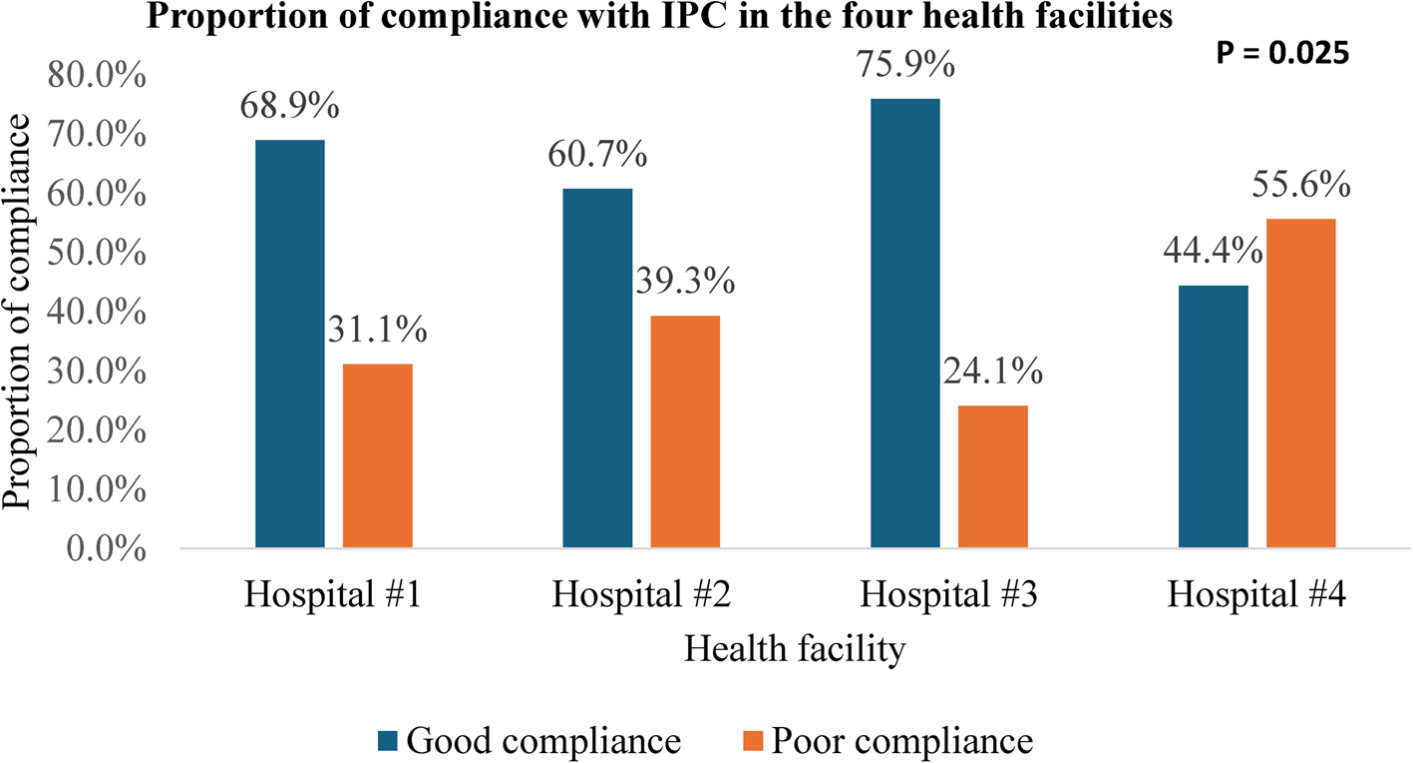
Proportion of compliance with IPC in four health facilities in Fako division, Cameroon

Concerning compliance with standard precautions per health facilities, Hospital #3 had a higher proportion of compliance (75.9%), followed by Hospital #1 (68.9%), Hospital #2 (60.7%), and Hospital #4 (44.4%) (Fig. 1).

### Compliance with IPC by cadre

**Fig. 2:**
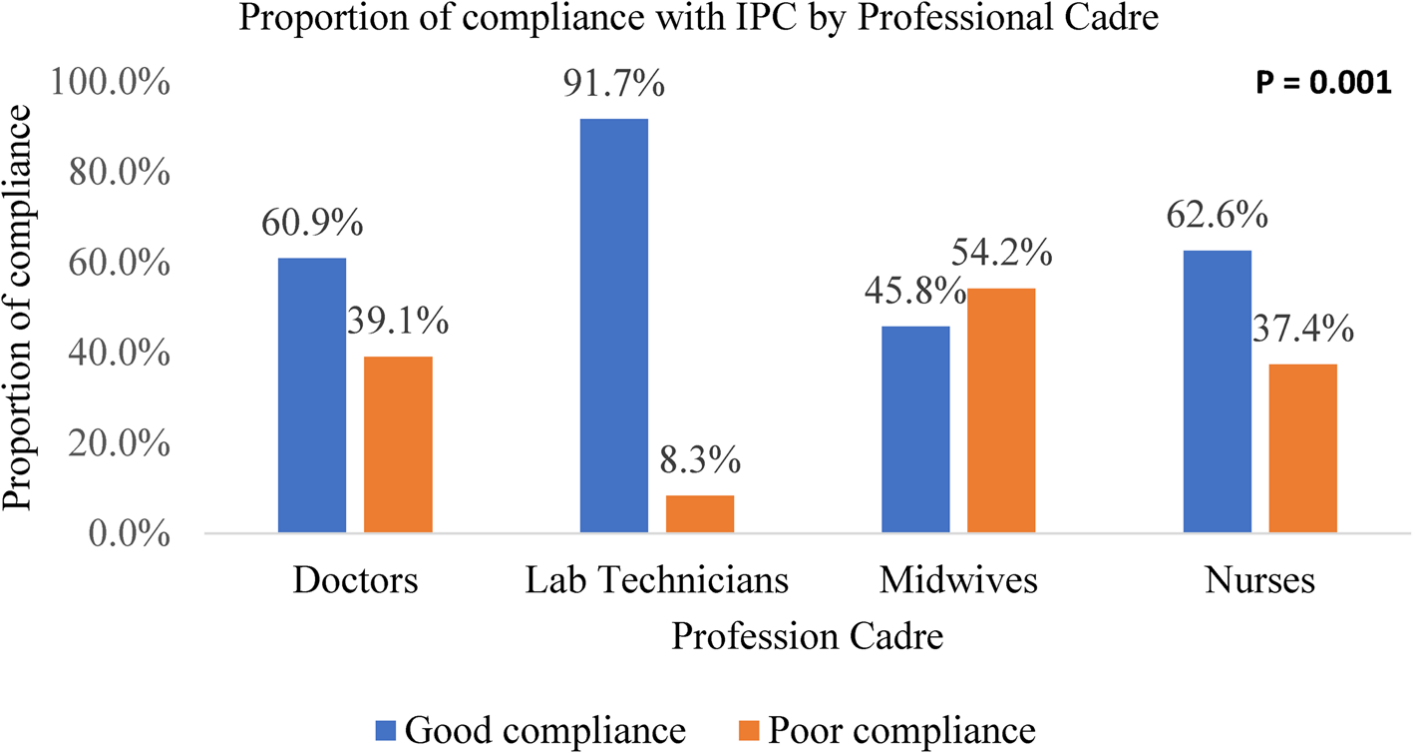
Proportion of compliance with IPC according to Professional Cadre in four health facilities in Fako division, Cameroon

Laboratory technicians had a compliance to IPC proportion of 91.7%, nurses 62.4%, doctors 60.9%, and midwives 45.8% (Fig. 2).

### Compliance with IPC by department

**Fig. 3:**
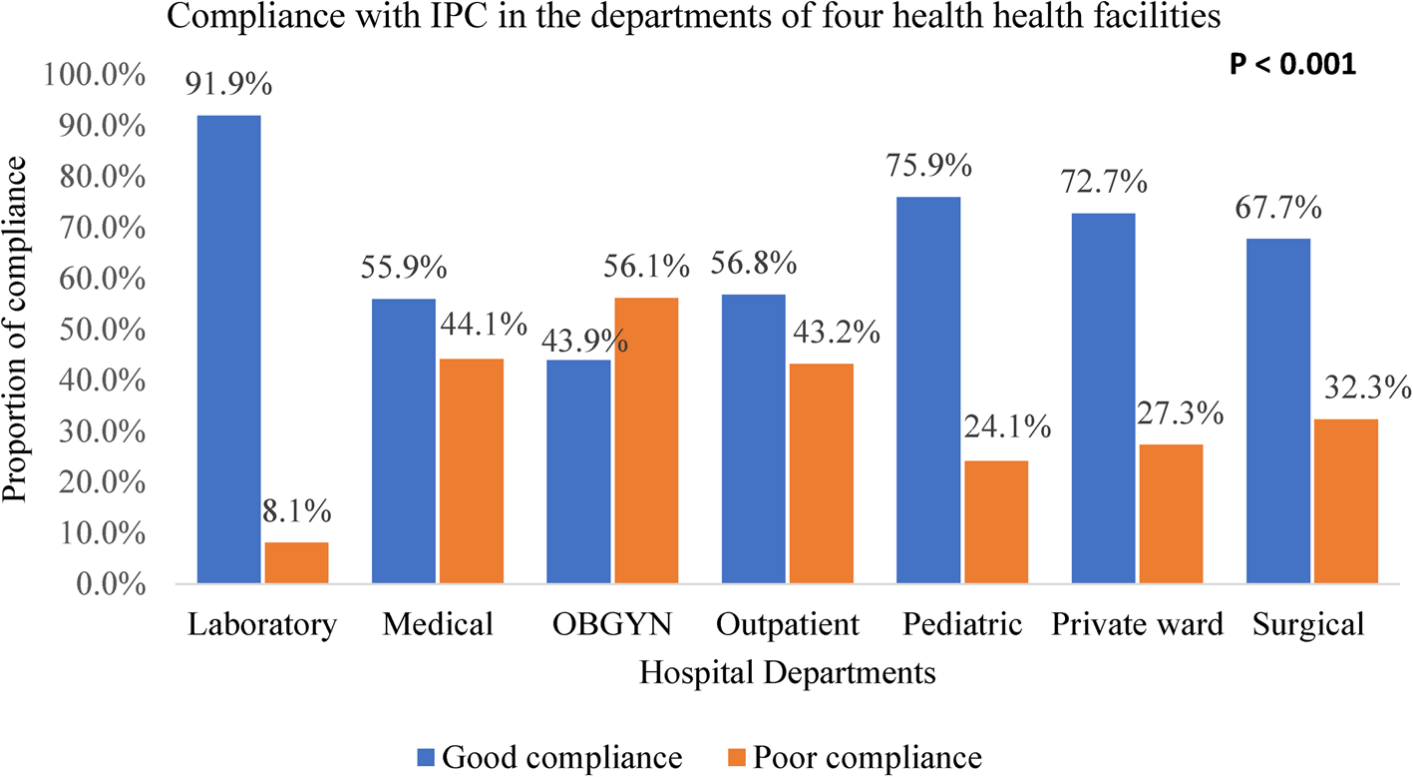
Proportion of compliance with IPC in the departments of four health facilities in Fako division, Cameroon

Among departments, the Laboratory department had a compliance proportion to IPC of 91.9%, the Pediatric department 75.9%, the Private ward 72.7%, the Surgical department 67.7%, the Outpatient department 56.8%, the Medical department 55.9%, and the OBGYN department 43.9% (Fig. 3).

### Factors independently associated with good compliance with IPC among healthcare workers

In the multivariable analysis, three variables were independently associated with good compliance with SPs. For the professional cadre, the odds of laboratory scientists practicing good compliance were 8.32 times higher [aOR = 8.32 (95%CI:1.90–36.53), *P* = 0.005] compared to midwives. The odds of Hospital #3 having good compliance were 3.61 times higher [aOR = 3.61 (95%CI; 1.29–10.07), *P* = 0.014] compared to Hospital #4. The odds of good compliance were 2.41 times higher [aOR = 2.41 (95%: 1.38–4.19), *P* = 0.002] when transmission-based precaution was needed compared to when it was not needed (Table 3).

**Table 3:** Factors associated with IPC compliance among healthcare workers in four health facilities in Fako division, Cameroon

**Variables**	**Univariable analysis (*n* = 276)**				** Multivariable analysis (*n* = 276)**			
	**%**	**OR**	**(95%CI)**	***P*-value**	**%**	**aOR**	**(95%CI)**	*P*-value*
**Gender**								
Male	27.2	1.91	(1.06 - 3.45)	**0.032**	27.2	1.95	(0.98– 3.89)	0.054
Female		1			72.8	1		
**Age (in years)**								
18 to ≤ 25	81.9	0.20	(0.03 - 1.66)	0.137	81.9	0.39	(0.04 - 3.94)	0.428
> 25 to ≤ 35	14.9	0.34	(0.04 - 3.05)	0.336	14.9	0.56	(0.05 - 5.87)	0.631
> 35	3.3	1			3.3	1		
**Cadre**								
Doctors	25.0	1.84	(0.72 - 4.69)	0.203	25.0	2.27	(0.80 - 6.44)	0.124
Lab. Technicians	13.0	13.00	(3.11 - 54.26)	**<0.001**	13.0	8.32	(1.90 - 36.53)	0.005
Nurses	53.3	1.98	(0.83 - 4.72)	0.125	53.3	1.72	(0.69 - 4.31)	0.248
Midwives	8.7	1			8.7	1		
**Work status**								
Contract	23.9	1.91	(0.75 - 4.88)	0.176	23.9	2.02	(0.69 - 5.95)	0.199
Volunteer	65.6	0.95	(0.42 - 2.12)	0.894	65.6	0.84	(0.34 - 2.07)	0.703
State worker	10.5	1			10.5	1		
**Facility**								
Hospital #1	26.8	2.78	(1.12 - 6.85)	**0.027**	26.8	2.17	(0.84 - 5.62)	0.110
Hospital #2	42.4	1.93	(0.83 - 4.49)	0.127	42.4	1.62	(0.63 - 4.16)	0.318
Hospital #3	21.0	3.93	(1.49 - 10.35)	**0.006**	21.0	3.61	(1.29 - 10.07)	0.014
Hospital #4	9.8	1			9.8	1		
**PPE**								
Yes	87.3	1.63	(0.80 - 3.34)	0.180	87.3	1.05	(0.44 - 2.52)	0.913
No	12.7	1				1		
**Needle stick exposure**								
Yes	74.3	1.36	(0.78 - 2.37)	0.276				
No	25.7	1						
**IPC guideline**								
Yes	87.0	2.00	(0.99 - 4.05)	0.054	87.0	1.57	(0.72 - 3.46)	0.260
No		1				1		
**IPC training**								
Yes	67.7	1.11	(0.65 - 1.87)	0.707				
No		1						
**Constant electricity**								
Yes	80.8	1.83	(1.00 - 3.37)	0.050	80.8	1.67	(0.84 - 3.28)	0.142
No		1				1		
**TBP needed**								
Yes	51.5	2.71	(1.63 - 4.52)	**<0.001**	51.5	2.44	(1.36 - 4.38)	**0.002**
No		1			48.5	1		
**IPC Knowledge**								
Good knowledge	37.7	0.71	(0.43- 1.18)	0.442				
Poor knowledge		1						

*PPE* Personal protective equipment, *IPC* Infection prevention and control, *TBP* Transmission-based precautions, *OR* Odds ratio, *aOR* Adjusted odds ratio, n= sample, *%* Percentage, *CI* Confidence interval

## Discussion

This study aimed to assess healthcare workers’ compliance with infection prevention control standard precaution measures and its associated factors. Overall, approximately 13 out of 20 HCWs had good compliance with IPC. Professional cadre(lab.technician), facility(Hospital #3), and the need for transmission-based precautions were independently associated with good compliance with IPC measures.

The compliance proportion with IPC of 64.5% in our study was congruent with what was obtained in similar studies conducted in Ghana in 2022 (65.6%) [15] and in Ethiopia in 2021 (57.8%) [16]. However, this is higher than the compliance rate of 34.49% reported in a study conducted by Senbato et al., 2024 in Addis Ababa [13]. This discrepancy can be explained by the fact that in their study, optimal compliance was set at a stricter score of > 85% compared to the score of > 80% in our study. Concerning compliance per stratum of the profession, laboratory technicians had the highest. This high level of compliance among laboratory technicians can be explained by their higher level of knowledge and a higher risk of exposure to HAI.

Our study showed that Cadre, health facility, and the need for transmission-based precaution were independently associated with good compliance with IPC. We found that the odds of better compliance among laboratory technicians was 13.0 compared to the midwives. These higher odds of compliance among laboratory technicians can be explained by the fact that in our study population, laboratory technicians had the highest proportion of compliance (91.7%). A similar study conducted in Northern Ethiopia found that compared to laboratory technicians, doctors, and nurses had 80% and 70% reduced odds of good practice, respectively [17]. Contrary to our study, a study conducted in Tanzania in 2024 found that nurses were more likely to comply with IPC compared to other cadre of healthcare workers [18]. According to that study, nurses were found to have IPC training in their educational curriculum, which they applied in practice.

Our study found that compliance varied significantly by health facilities with Hospital #3 showing the highest compliance rate. Moreover, Hospital #3 had an IPC committee with regular monthly meetings with staff to discuss IPC compliance. This is similar to the finding of Alhumaid et al., 2021 where HCW’s participation in the IPC committee improved adherence to IPC measures [19].

The need for transmission-based precaution was statistically significantly associated with compliance with IPC. This could be explained by the fact that transmission-based precautions are applied in patients known or suspected to be colonised or infected with highly transmissible or epidemiologically significant pathogens [20]. This knowledge will, therefore, have a significant positive impact on good compliance with IPC.

In our study, knowledge of IPC was not statistically significantly associated with good compliance with SPs. This could be explained by the fact that in our study, only 34.8% of the population had good knowledge of IPC. Similarly, a study conducted in Ghana in 2022 concluded that knowledge of IPC doesn’t influence compliance with SPs [15]. This is because knowledge alone does not necessarily translate into good practice but requires consciousness and other personal facility-level and policy that will enable standard practice. However, a study conducted by Adil Aboulkhail and Thamer Alslamah in 2022 stated that a lack of knowledge of the recommended practices hinders compliance with IPC [21].

### Study strength

In our study, we used an observation form which is standard according to WHO to evaluate the compliance of healthcare workers.

### Study limitations

Our study may have experienced the Hawthorne effect, with the participants possibly adjusting their practice because they were being observed. However, we tried to mitigate this effect by allowing at least 24 h to elapse from the time of signing of the informed consent form to participant observation. We also reduced this effect by administering the structured questionnaire only after participant observation.

Furthermore, our study did not include students who were involved in the clinical and paraclinical aspects of patient care including infection control. This excluded a good proportion of the workforce in the health facilities who were exposed to infection and likely to transmit HAI.

## Conclusion

The compliance with infection prevention control standard precaution measures of HCWs in the Fako Division is suboptimal and varies with the professional qualification, health facilities, and departments. Factors associated with good compliance have been identified. These findings highlight the need for hospital-based interventions to improve HCW compliance with IPC measures and to break the infection transmission chain in the hospitals. To curb the burden of HAI, we recommend that health facilities implement regular IPC compliance follow-ups through IPC committees to reinforce and sustain compliance with IPC measures. HCWs should make a conscious effort to participate in IPC training and make good use of the available guidelines to adhere to IPC measures.

## Supplementary Information

Supplementary Material 1.

Supplementary Material 2.

## Data Availability

All data from the results of this study are available upon reasonable request from the corresponding author.

## Abbreviations

HAI: Healthcare-associated infection
HBV: Hepatitis B Virus
HCV: Hepatitis C Virus
HCW: Healthcare worker
HIV: Human immunodeficiency Virus
ICU: Intensive Care Unit
IPC: Infection Prevention and Control
LMIC: Low and middle-income countries
PPE: Personal protective equipment
SP: Standard precautions
SP: Standard precautions
SSA: Sub-Saharan Africa
TBP: Transmission-based precautions
UP: Universal precautions
WHO: World Health Organization

## References

[1] Gidey K, Gidey MT, Hailu BY, Gebreamlak ZB, Niriayo YL. Clinical and economic burden of healthcare-associated infections: A prospective cohort study. Do Prado PR. editor PLoS ONE. 2023;18(2):e0282141. 10.1371/journal.pone.0282141 PMC9949640 36821590

[2] Abubakar U, Amir O, Rodríguez-Baño J. Healthcare-associated infections in Africa: a systematic review and meta-analysis of point prevalence studies. J Pharm Policy Pract. 2022;15:99. 36494700 10.1186/s40545-022-00500-5 PMC9733066

[3] Nouetchognou JS, Ateudjieu J, Jemea B, Mesumbe EN, Mbanya D. Surveillance of nosocomial infections in the Yaounde University Teaching Hospital, Cameroon. BMC Res Notes. 2016;9(1):505. 27931241 10.1186/s13104-016-2310-1 PMC5146876

[4] Ndu AC, Arinze-Onyia SU. Standard precaution knowledge and adherence: Do Doctors differ from Medical Laboratory Scientists? Mal Med J. 2018;29(4):294. 10.4314/mmj.v29i4.3 PMC6019545 29963283

[5] Yasin J, Fisseha R, Mekonnen F, Yirdaw K. Occupational exposure to blood and body fluids and associated factors among health care workers at the University of Gondar Hospital, Northwest Ethiopia. Environ Health Prev Med. 2019;24(1):18. 30851726 10.1186/s12199-019-0769-9 PMC6408855

[6] Tomczyk S, Twyman A, De Kraker MEA, Coutinho Rehse AP, Tartari E, Toledo JP, Cassini A, Pittet D, Allegranzi B. The first WHO global survey on infection prevention and control in health-care facilities. Lancet Infect Dis. 2022;22(6):845–56. 35202599 10.1016/S1473-3099(21)00809-4 PMC9132775

[7] National guidelines on infection prevention and control in health facilities in Cameroon [Internet]. CCOUSP. [cited 2025 Jan 13]. Available from: https://www.ccousp.cm/download/national-guidelines-on-infection-prevention-and-control-in-health-facilities-in-cameroon/.

[8] Broussard IM, Kahwaji CI. Universal Precautions. In: StatPearls. Treasure Island (FL): StatPearls Publishing; 2023 [cited 2023 Oct 1]. Available from: http://www.ncbi.nlm.nih.gov/books/NBK470223/. 29262198

[9] Siegel et al. 2007 Guideline for Isolation Precautions: Preventing Transmission of Infectious Agents in Healthcare Settings. [cited 2023 Oct 1]. Available from: https://www.cdc.gov/infection-control/hcp/isolation-precautions/index.html. 10.1016/j.ajic.2007.10.007 PMC7119119 18068815

[10] Compliance with Universal. /Standard Precautions among health care workers in rural north India - ScienceDirect. [cited 2023 Aug 27]. Available from: https://www.sciencedirect.com/science/article/abs/pii/S0196655304006133.

[11] Nofal M, Subih M, Al-Kalaldeh M, Al Hussami M. Factors influencing compliance to the infection control precautions among nurses and physicians in Jordan: A cross-sectional study. J Infect Prev. 2017;18(4):182–8. 28989525 10.1177/1757177417693676 PMC5496691

[12] Sh H, Wm E, Es M, Fe M, Knowledge. Attitude and Practice of Infection Prevention Measures among Health Care Workers in Wolaitta Sodo Otona Teaching and Referral Hospital. J Nurs Care. 2017;06(04). [cited 2024 May 31]. Available from: https://www.omicsonline.org/open-access/knowledge-attitude-and-practice-of-infection-prevention-measures-among-health-care-workers-in-wolaitta-sodo-otona-teaching-and-ref-2167-1168-1000416.php?aid=92509.

[13] Senbato FR, Wolde D, Belina M, Kotiso KS, Medhin G, Amogne W, Eguale T. Compliance with infection prevention and control standard precautions and factors associated with noncompliance among healthcare workers working in public hospitals in Addis Ababa, Ethiopia. Antimicrob Resist Infect Control. 2024;13(1):32. 38475931 10.1186/s13756-024-01381-w PMC10935924

[14] Hand hygiene technical. reference manual: to be used by health-care workers, trainers and observers of hand hygiene practices. [cited 2024 Sep 24]. Available from: https://www.who.int/publications/i/item/9789241598606.

[15] Knowledge. and determinants of infection prevention and control compliance among nurses in Yendi municipality, Ghana| PLOS ONE. [cited 2024 Sep 24]. Available from: https://journals.plos.org/plosone/article?id=10.1371/journal.pone.0270508. 10.1371/journal.pone.0270508 PMC9299325 35857742

[16] Limenyande MJM, Kobusingye JO, Tindyebwa T, Akongo D, Isunju JB, Musoke D. Factors associated with compliance with Infection Prevention and Control measures during the COVID-19 pandemic among healthcare workers in Kampala City, Uganda. PLoS ONE. 2023;18(11):e0293732. 37910487 10.1371/journal.pone.0293732 PMC10619793

[17] Yemane D, Standard Precautions Practice among Health Care Workers in Public Health Facilities of Mekelle Special Zone, Northern Ethiopia. J Community Med Health Educ. 2014;04(03). [cited 2024 Sep 5]. Available from: https://www.omicsonline.org/open-access/standard-precautions-practice-among-health-care-workers-in-public-health-facilities-of-mekelle-special-zone-northern-ethiopia-2161-0711.1000286.php?aid=26319.

[18] Edward M, John W, Mahulu E, Lyabangi JF, Nkumba O, et al. Challenges of compliance with infection prevention and control (IPC) standard procedures among healthcare workers: a hospital-based cross-sectional study. Int J Health Policy Plan. 2024;3(1):01–07.

[19] Alhumaid S, Al Mutair A, Al Alawi Z, Alsuliman M, Ahmed GY, Rabaan AA, Al-Tawfiq JA, Al-Omari A. Knowledge of infection prevention and control among healthcare workers and factors influencing compliance: a systematic review. Antimicrob Resist Infect Control. 2021;10(1):86. 34082822 10.1186/s13756-021-00957-0 PMC8173512

[20] National Infection Prevention and Control Guidelines-Sierra Leone [Internet]; 2015. [cited 2024 Jan 7]. Available from: https://www.afro.who.int/sites/default/files/2017-05/ipcguide.pdf.

[21] Abalkhail A, Alslamah T. Institutional Factors Associated with Infection Prevention and Control Practices Globally during the Infectious Pandemics in Resource-Limited Settings. Vaccines (Basel). 2022;10(11):1811. 36366320 10.3390/vaccines10111811 PMC9696365

[22] WMA - The World Medical. Association-WMA Declaration of Helsinki– Ethical Principles for Medical Research Involving Human Participants. [cited 2025 Mar 12]. Available from: https://www.wma.net/policies-post/wma-declaration-of-helsinki/. 10.1001/jama.2024.21972 39425955

