# Supplementary material for: Compliance with infection prevention and control standard precautions and associated factors among healthcare workers in four health facilities in Fako division, Cameroon
Source: BMC Health Serv Res. 2025 Apr 24;25:596. doi: 10.1186/s12913-025-12594-z (PMC12023434; doi:10.1186/s12913-025-12594-z)
Supplement: Supplementary file 1 — Supplementary Material 1. [file 12913_2025_12594_MOESM1_ESM.docx]

**Compliance Observation form**

| Facility: | Date : |
| --- | --- |
| Ward: | Start time: |
| Shift: morning 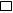 night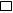 | End time: |
|  | Session duration: |

| Prof.cat | |  | | |
| --- | --- | --- | --- | --- |
| code | |  | | |
| N | |  | | |
| **Opportunity** | **Indication** | | **HH Action** | **Specific disease (s):……………** |
| 1 | Before touching patient. 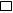  Before-aseptic procedure. 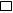  After body fluid exposure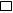 risk.  After touching a patient. 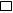  After touching patient 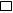surrounding | | **Hand Rub**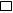  **Hand Washing**  🌕 missed  **🌕 gloves** | **Face Mask**  🌕 missed  **Goggle**  🌕 missed  **Gown** or **apron**  🌕 missed |
| 2 | Before touching patient. 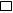  Before-aseptic procedure. 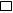  After body fluid exposure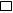 risk.  After touching a patient. 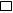  After touching patient 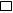surrounding | | **Hand Rub**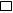  **Hand Washing**  🌕 missed  **🌕 gloves** | **Face Mask**  🌕 missed  **Goggle**  🌕 missed  **Gown** or **apron**  🌕 missed |
| 3 | Before touching patient. 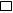  Before-aseptic procedure. 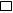  After body fluid exposure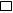 risk.  After touching a patient. 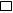  After touching patient 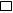surrounding | | **Hand Rub**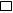  **Hand Washing**  🌕 missed  **🌕 gloves** | **Face Mask**  🌕 missed  **Goggle**  🌕 missed  **Gown** or **apron**  🌕 missed |

*NB: Glove use may be recorded only when the hand hygiene action is missed while the health-care worker is wearing gloves.*
