# Supplementary material for: Compliance with infection prevention and control standard precautions and associated factors among healthcare workers in four health facilities in Fako division, Cameroon
Source: BMC Health Serv Res. 2025 Apr 24;25:596. doi: 10.1186/s12913-025-12594-z (PMC12023434; doi:10.1186/s12913-025-12594-z)
Supplement: Supplementary file 2 — Supplementary Material 2. [file 12913_2025_12594_MOESM2_ESM.docx]

**Knowledge of Infection Prevention and Control Questionnaire**

| **S/N** | **Question** | **Answers** |
| --- | --- | --- |
| 1 | Hand washing is necessary before and after procedures are performed. | 1. Yes 2. No |
| 2 | Gloves provide complete protection against  transmission of infections. | 1. Yes 2. No |
| 3 | All needles should be recapped after injection | 1. Yes 2. No |
| 4 | Is the use of an alcohol-based antiseptic for hand hygiene effective as soap and water if hands are not visibly dirty? | 1. Yes 2. No |
| 5 | Gloves should be worn if blood or body fluid exposure is anticipated | 1. Yes 2. No |
| 6 | Should waste be segregated at the point of generation? | 1. Yes 2. No |
| 7 | Is tuberculosis (TB) carried in airborne particles that are generated from patients with active pulmonary tuberculosis? | 1. Yes 2. No |
| 8 | Is there a need to change gloves between patients as  long as there is no visible contamination? | 1. Yes 2. No |
| 9 | Do you know how to prepare 0.5% chlorine solution? | 1. Yes 2. No |
| 10 | Safety box should be used when three quarters full. | 1. Yes 2. No |
